# Protective effect of Shenmai injection on doxorubicin-induced cardiotoxicity via regulation of inflammatory mediators

**DOI:** 10.1186/s12906-019-2686-2

**Published:** 2019-11-19

**Authors:** Sheng Zhang, Zhen-Qiang You, Lin Yang, Li-Li Li, You-Ping Wu, Li-Qiang Gu, Yan-Fei Xin

**Affiliations:** 1 Center of Safety Evaluation, Zhejiang Academy of Medical Sciences, 182 Tianmushan Road, Hangzhou, 310013 Zhejiang China; 2 Collaborative Innovation Center of Yangtze River Delta Region Green Pharmaceuticals, Zhejiang University of Technology, Hangzhou, Zhejiang China; 3 The Children’s Hospital Zhejiang University School of Medicine, Hangzhou, Zhejiang China

**Keywords:** Doxorubicin, Shenmai injection, Cardiotoxicity, Anti-inflammatory

## Abstract

**Background:**

Doxorubicin (DOX) is a chemotherapy drug for malignant tumors. The clinical application of DOX is limited due to its dosage relative cardiotoxicity. Oxidative damage and cardiac inflammation appear to be involved in DOX-related cardiotoxicity. Shenmai injection (SMI), which mainly consists of *Panax ginseng*C.A.Mey.and *Ophiopogon japonicus* (Thunb.) Ker Gawl*,* is widely used for the treatment of atherosclerotic coronary heart disease and viral myocarditis in China. In this study, we investigated the protective effect of Shenmai injection on doxorubicin-induced acute cardiac injury via the regulation of inflammatory mediators.

**Methods:**

Male ICR mice were randomly divided into seven groups: control, DOX (10 mg/kg), SMI (5 g/kg), DOX with pretreatment with SMI (0.5 g/kg, 1.5 g/kg or 5 g/kg) and DOX with post-treatment with SMI (5 g/kg). Forty-eight hours after the last DOX administration, all mice were anesthetized for ultrasound echocardiography. Then, serum was collected for biochemical and inflammatory cytokine detection, and heart tissue was collected for histological and Western blot detection.

**Results:**

A cumulative dose of DOX (10 mg/kg) induced acute cardiotoxicity in mice manifested by altered echocardiographic outcome, and increased tumor necrosis factor, interleukin 6 (IL-6), monocyte chemotactic protein 1, interferon-γ, and serum AST and LDH levels, as well as cardiac cytoplasmic vacuolation and myofibrillar disarrangement. DOX also caused the increase in the expression of IKK-α and iNOS and produced a large amount of NO, resulting in the accumulation of nitrotyrosine in the heart tissue. Pretreatment with SMI elicited a dose-dependent cardioprotective effect in DOX-dosed mice as evidenced by the normalization of serum inflammatory mediators, as well as improve dcardiac function and myofibril disarrangement.

**Conclusions:**

SMI could recover inflammatory cytokine levels and suppress the expression of IKK-α and iNOS in vivo, which was increased by DOX. Overall, there was evidence that SMI could ameliorate DOX-induced cardiotoxicity by inhibiting inflammation and recovering heart dysfunction.

## Background

Doxorubicin (DOX), an anthraquinone antibiotic for cancer therapy, is widely used in clinic [1, 2]. However, DOX has severe cardiotoxic side effects, limiting its clinical application. Studies have shown that when the cumulative dose of DOX reaches more than 500 mg/m^2^, the risk of fatal cardiomyopathy increases. Furthermore, DOX leading to progressive and dose-related cardiotoxicity has been reported [3]. In addition, DOX-induced cardiomyopathy (DIC) has clinical features with poor prognosis and high mortality [4]. Moreover, the use of DOX is another important cause of dilated cardiomyopathy that causes congestive heart failure.

Doxorubicin-induced cardiotoxicity is considered as multifactorial. Extensive evidence has provided putative mechanisms; nevertheless, the precise mechanism underlying DOX-induced cardiotoxicity is still not completely elucidated [5]. Although basic research has shown that doxorubicin-induced cardiotoxicity mechanisms have many classes [6]. Traditionally, most studies focuses on reactive oxygen species (ROS). Indeed, numerous studies have shown that cardiac inflammation due to ROS production and the subsequent apoptosis of cardiomyocytes is widely accepted as the main mechanism of DIC [7]. Subsequently, pro-inflammatory cytokines, inflammatory cell infiltration, and necrosis in cardiac tissues are increased [8]. Inflammation and cell death lead to progressive cardiomyocyte injury and cellular loss, resulting in the thinning of ventricular walls and decreased systolic performance [9].

Shenmai injection (SMI), which mainly consists of *Panax ginseng*C.A.Mey.and *Ophiopogon japonicus* (Thunb.) Ker Gawl, is commonly used in coronary heart disease and chronic pulmonary heart disease treatment [10]. Ginsenosides have been identified as the most important active ingredient in SMI [11]. SMI can inhibit lipid oxidation by scavenging oxygen-derived radicals [10]. Furthermore, most studies have focused on SMI for improving the immune function of cancer patients and decreasing the inflammatory mediators released by innate immune cells [12]. We hypothesized that SMI could effectively inhibit DOX-induced cardiotoxicity via regulating the innate immune response. In this study, we investigated SMI effects against DOX-induced cardiotoxicity and elucidated the underlying mechanisms of inflammatory mediators via the activation of the nuclear factor Kappa-B (NF-κB) pathway. In addition, the expression of inducible nitricoxide synthase (iNOS) was increased in cardiomyocytes in response to high levels of cytosolic nitric oxide (NO), which lead to the release of pro-inflammatory mediators by innate immune cells [13].

## Methods

### Materials

Doxorubicin was obtained from Wuhan far co Creation Technology Co., Ltd. (Wuhan, China) and dissolved in 0.9% saline for injection. Shenmai injection, 10 mL per bottle, was provided by Chiatai Qing Chun Bao Pharmaceutical Co., Ltd. (Hangzhou, China). One bottle of SMI contains 2 g of crude drugs (1 g of *Panax ginseng*C.A.Mey. and 1 g of *Ophiopogonjaponicus* (Thunb.) Ker Gawl.). Where not indicated otherwise, the products were purchased from Sangon Biotech (Shanghai, China) Co., Ltd.

### UHPLC analysis of ginsenosides in SMI

**Fig. 1:**
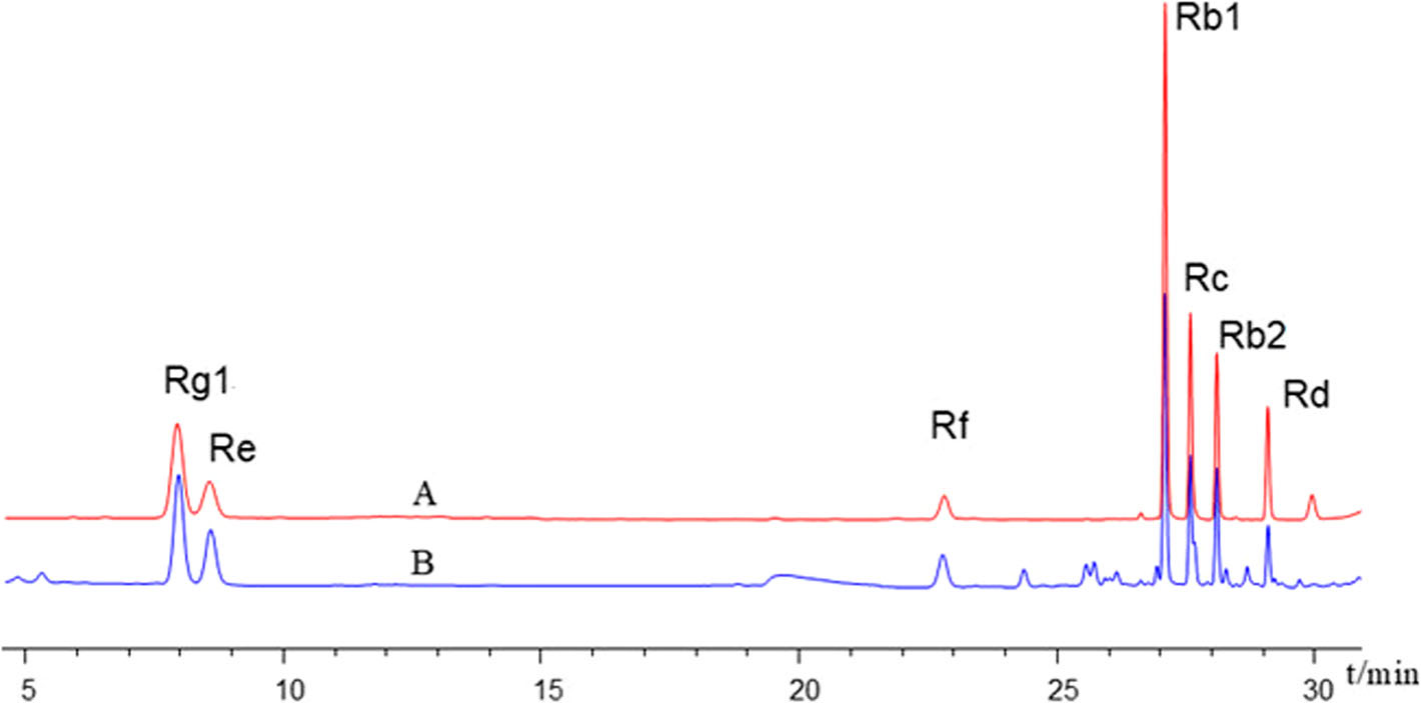
UHPLC chromatogram of standard product of ginsenosides (A) and Shenmai Injection (B) used in the present study

The standard products of ginsenosides (Rg1, Re, Rf, Rb1, Rc, Rd., and Rb2) were purchased from Shanghai Yuanye Bio-Technology Co., Ltd. (Shanghai, China). UHPLC (Agilent Technologies, Santa Clara, USA) was employed to achieve the simultaneous detection of 7 main kinds of ginsenosides (Rg1, Re, Rf, Rb1, Rc, Rd., and Rb2) in SMI. Excellent separation of analytes was achieved using an Agilent Eclipse plus column (50 mm × 2.1 mm, 1.8 μm). The gradient elution system consisted of water (A) and acetonitrile (B) of 0.3 mL/min. A gradient elution program was used as follows: 0–10 min, 19% B; 10–18 min, 19–23% B; 18–21 min, 23% B; and 21–31 min, 23–40% B. The UV detection wavelength was set at 203 nm, and the injection volume was 1 μL. Retention time is shown in Fig. 1. The ginsenoside concentrations of the samples were quantified against standard curves. The contents of ginsenosides in the SMI were as follows: Rg1, 0.16 mg/mL; Re, 0.08 mg/mL; Rf, 0.05 mg/mL; Rb1, 0.17 mg/mL; Rc, 0.05 mg/mL; Rd., 0.02 mg/mL; and Rb2, 0.06 mg/mL.

### Animal

Seventy male specific pathogen-free ICR mice weighing approximately 22–25 g were purchased from Shanghai Slack Laboratory Animal Co., Ltd. The mice were housed in microisolator cages and given ad libitum access to food and water. All mice were housed in a barrier system, a clean environment with a temperature of 20 to 25 °C and a humidity of 50 to 60%, 12-h bright and 12-h dark lighting cycle was for life light. Animal experiments were conducted in laboratories which passed the authentication of the Association for Assessment and Accreditation of Laboratory Animal Care. The general observations were detected during the experimental days, including animal profile, weight, livability and so on. At the end of the experiment, all of the mice were anesthetized with 3% of isoflurane. When the rats were completely anesthetized, Orbital blood was collected by capillary, and the mice were sacrificed by cervical vertebra dislocation.

### Experimental protocol

**Fig. 2:**
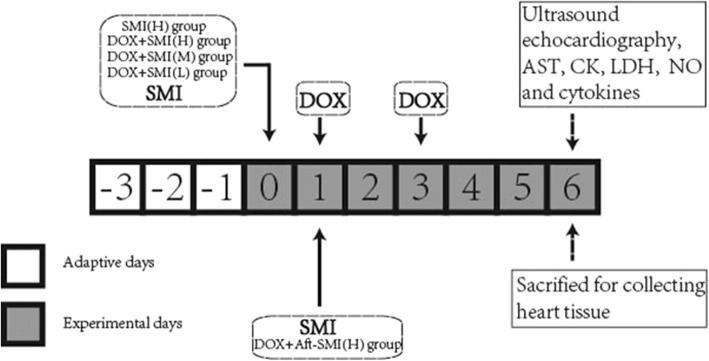
The experimental protocol timeline

After 3 days of adaptation to the housing environment, the mice were randomly divided into seven groups: control group (Control), doxorubicin-induced group (10 mg/kg, DOX), SM injection group (5 g/kg, SMI (H)), co-treatment with DOX and SMI (0.5 g/kg) group (DOX + SMI(L)), co-treatment with DOX and SMI (1.5 g/kg) group (DOX + SMI(M)), co-treatment with DOX and SMI (5 g/kg) group (DOX + SMI(H)) and cotreatment with DOX and SMI (5 g/kg) after treatment (DOX + Aft – SMI (H)). All mice treated with DOX in the experimental group received an intraperitoneal injection of DOX on the third experimental day. In the DOX + Aft – SMI (H) group, SMI was intravenously administered within 6 h after the first administration of DOX. In the other groups of patients treated with SMI, SMI was intravenously administered within 24 h before the first administration of DOX. The mice in the control group were injected with an equivalent volume of saline. The SMI (H) groups were i.p. injected with an equivalent volume of saline. The mortality rate and general appearance of the animals were observed and recorded. At 48 h after the last DOX administration, all the mice were received ultrasound echocardiography evaluation. At the end of the text of ultrasound echocardiography collection, mice were sacrificed by bloodletting under anesthesia, and blood samples were collected for the evaluation of serum levels of AST, CK and LDH. The hearts were removed for subsequent analyses. The experimental protocol is shown in Fig. 2.

### Ultrasound echocardiography evaluation in vivo

Cardiac function was evaluated by echocardiography. Animals were anesthetized using 3% inhalant isoflurane in 100% oxygen and maintained in 1.5–2% isoflurane during the echocardiogram testing. M-mode, B-mode, and left ventricular outflow tract (LVOT) ultrasound images were obtained by a Vevo 1100 ultrasound system (Visual Sonics, Toronto, Canada). The following parameters, including heart ratio (HR, BMP), cardiac output (CO mL/min), left ventricular ejection fraction percentage (EF %) and fractional shortening percentage (FS %), were measured as indicators of left ventricular function.

### Determination of serum AST, CK, LDH, and NO levels

The AST, CK and LDH enzyme activities in the serum were detected by a Hitachi automatic analyzer (Model 7100, Japan). NO content in serum is assessed by NO assay kit (Nanjing Jiancheng Bioengineering Institute, Nanjing, China) under the instructions of manufacturer.

### Histopathologic examination

Heart tissue samples were fixed overnight in 10% formalin, dehydrated by serial concentrations of ethanol, and finally embedded in paraffin blocks. Cut the paraffin block into 5 μm slices and stain with hematoxylin and eosin. A pathologist blinded to the treatments performed the histopathologic examination with a microscope (Leica DM4000, Germany). Histopathologic sections were scored following the previous report [14]. The grading system was briefly described as follows: grade 0: no change, grade 1: < 5% of myofibrillar loss, grade 2: 5–30% of myofibrillar loss, grade 3: > 30% of myofibrillar loss.

### Cytokine determination

Pro-inflammatory cytokines, such as tumor necrosis factor (TNF), interleukin 6 (IL-6), monocyte chemotactic protein 1 (MCP-1) and Interferon-γ (INF-γ), in serum were measured by a mouse cytometric bead array Kit (BD Biosciences, USA). Briefly, 50 μL of homogenate supernatants was added to a mixture of 50 μL each of capture antibody bead reagent and phycoerythrin-conjugated detection antibody. Then the mixture was incubated at room temperature for 2 h in dark, and washed to remove unbound detection antibody as follow. Data were acquired using a BD FACSCalibur™ Flow Cytometer (BD Bioscience, San Jose, USA).

### Immunofluorescence

We detected the content of nitrotyrosine in frozen sections by immunofluorescence technique, and the mouse heart tissue was embedded in OCT compound (Bio-Optica, Milan, Italy). The 7 um tissue sections were prepared and incubated with rabbit anti-nitrotyrosine (R&D, USA) for 2 h at room temperature. After washing with PBS, the fluorescence in-conjugated secondary antibody was added for 1 h. finally the nuclei were counterstained with DAPI. The fluorescently stained slides were observed under an Olympus fluorescence microscope (Tokyo, Japan), and fluorescent images were taken.

### Immunoblot

Heart tissue was lysed with RIPA buffer (Beyotime Biotechnology, Shanghai, China). Protein concentrations of lysates were determined by BCA kits (Nanjing Jiancheng Bioengineering Institute, Nanjing, China). Equal amounts of protein (30 μg) from homogenized heart tissues were performed on SDS-polyacrylamide gels and transferred to PVDF membrane. Membranes were blocked with 5% fried skimmed milk for reduce non-specific binding for 1 h, and then incubated with primary antibody anti-iNOS (CST, Beverly, USA), anti-IKK-α (CST, Beverly, USA), or anti-GAPDH (Santa Cruz Biotechnology, Italy) as indicated overnight at 4 °C. After washing 3 times by TBST, HRP binding secondary antibodies were incubated with PVDF membranes. Immunoreactive protein bands were detected by chemiluminescence using enhanced chemiluminescence reagents (ECL) in ChemiScope 6000 system (Qinxiang, Shanghai, China).

### Statistics

The data are expressed as the means ± S.D. and analyzed by one-way ANOVA and Tukey’s HSD test. A *P* value of < 0.05 was considered statistically significant.

## Results

### General observations

At the end of the experiment, all mice were alive. However, the mice in the DOX-treated groups appeared weak, with hair erection, a hunched posture and weight loss. As shown in Table 1, DOX also caused a significant decrease (*P* < 0.05) in heart and body weight. SMI, at a dose of 5 g/kg by pretreatment, could significantly improve the body weight lost by DOX (P < 0.05). However, heart weight did not show any trend of recovery. Ascites was present in the DOX, DOX + SMI (L), DOX + SMI (M), and DOX + Aft – SMI (H) groups but not in the control, SMI (H), and DOX + SMI (H) groups. Compared with the mice injected with DOX only, the volume of ascites in the DOX + SMI (H) group was obviously reduced (*P* < 0.05).

**Table 1:** Effect of SMI and / or DOX treatment on animal characteristics

Group	Ascites (mL)	First Body Weight (g)	Final Body Weight (g)	Heart Weight (g)	HW/BW (mg/g)
Control	0.00 ± 0.00^#^	23.24 ± 1.92	31.38 ± 1.57^#^	0.14 ± 0.02^#^	4.31 ± 0.49
DOX	1.43 ± 0.32^*^	23.15 ± 2.08	26.17 ± 9.08^*^	0.11 ± 0.03^*^	4.06 ± 0.87
SM	0.00 ± 0.00^#^	23.02 ± 2.36	31.12 ± 1.46^#^	0.13 ± 0.01^#^	4.28 ± 0.34
DOX + SMI(L)	1.35 ± 0.30^*^	23.09 ± 2.16	26.47 ± 1.38^*^	0.10 ± 0.01^*^	3.61 ± 0.19^*^
DOX + SMI(M)	0.89 ± 0.16^*#^	23.49 ± 1.88	25.80 ± 1.69^*^	0.09 ± 0.01^*^	3.65 ± 0.28^*^
DOX + SMI(H)	0.00 ± 0.00^#^	23.07 ± 2.32	27.69 ± 1.78^*#^	0.12 ± 0.02^*^	4.22 ± 0.29
DOX + Aft-SMI(H)	0.74 ± 0.62^*#^	23.79 ± 2.26	26.98 ± 2.67^*^	0.10 ± 0.02^*^	3.55 ± 0.41^*#^

All values are mean ± S.D. (*n* = 10). *, significantly different (*P* < 0.05) from respective values in the control group. #, significantly different (*P* < 0.05) from respective values in the DOX group

### Cardiac function parameters

**Fig. 3:**
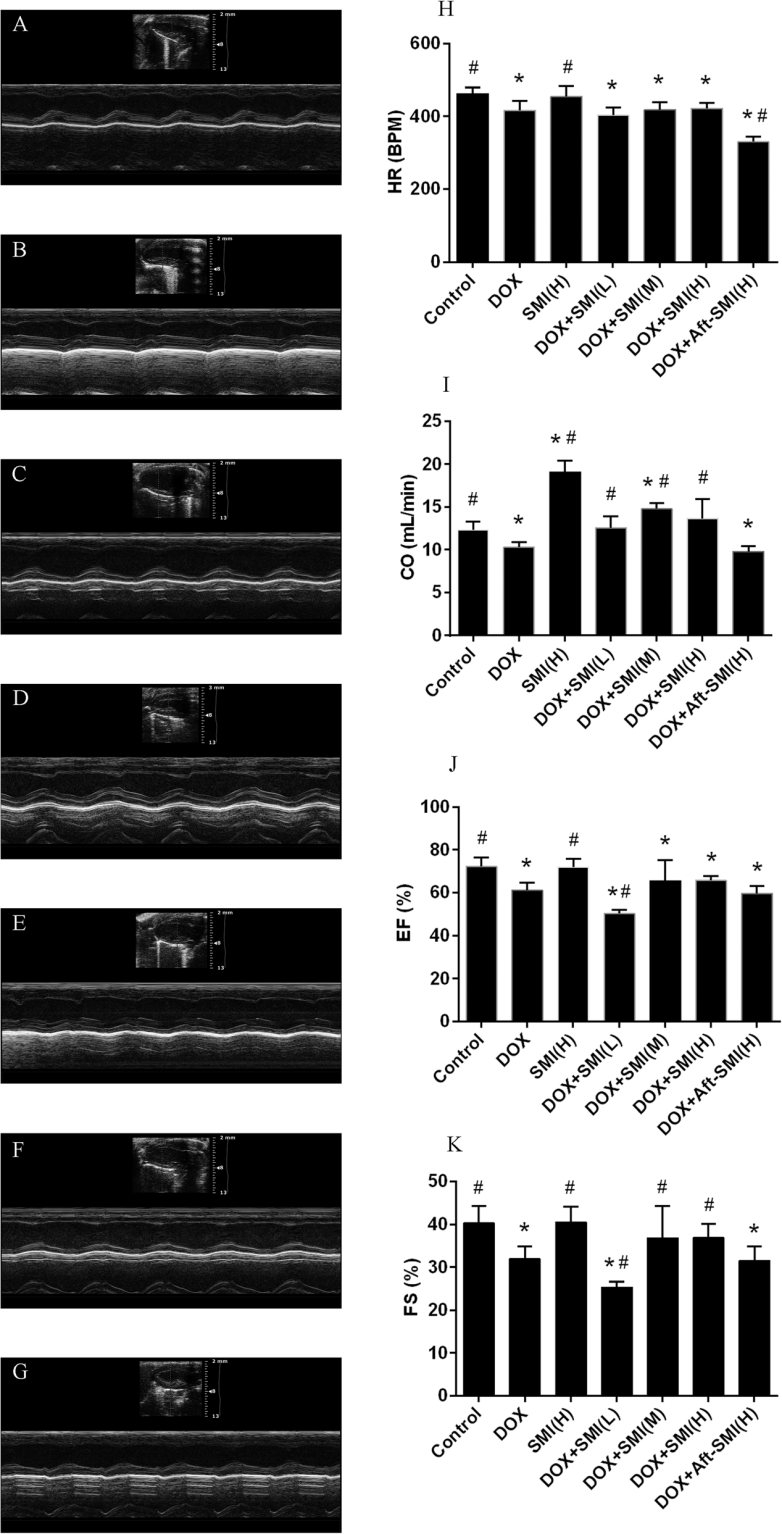
Effect of SMI on heart functional parameters after administration of DOX-inducedfrom control group (**a**), DOX group (**b**), SM injection group (**c**), co-treatment with DOX and SMI (0.5 g/kg) group (**d**), co-treatment with DOX and SMI (1.5 g/kg) group (**e**), co-treatment with DOX and SMI (5 g/kg) group (**f**) and the co-treatment with DOX and SMI (5 g/kg)) after treating (**g**). Representative M-mode echocardiograms of ICR mice. (**h**) Heart rate. (**i**) Cardiac output. (**j**) Ejection fraction. (**k**) Shortening index. The data are expressed as the mean ± S.D.; *n* = 10 in each group; *, significantly different (*P* < 0.05) from respective values in the control group; #, significantly different (*P* < 0.05) from respective values in the DOX group

Echocardiographic M-mode tracings and measurements were used to analyze heart function. Compared with the control group mice, the DOX-treated mice exhibited decreased heart function as evaluated by the heart rate (HR),cardiac output (CO ml/min), ejection fraction (EF %) and shortening index (FS %) (*P* < 0.05). As a result, cardiac functions were improved by SMI, which embodied CO and FS parameters in DOX + SMI (H) and DOX + SMI (M) Groups (Fig. 3h-k). Additionally, SMI can heighten myocardial function and increase cardiac output (*P* < 0.05) in normal mice. However, the data from the DOX + Aft – SMI (H) group showed that SMI administered after DOX treatment had a limited protective effect against cardiac function.

### Effects of SMI and/or DOX treatment on serum biochemistry

**Fig. 4:**
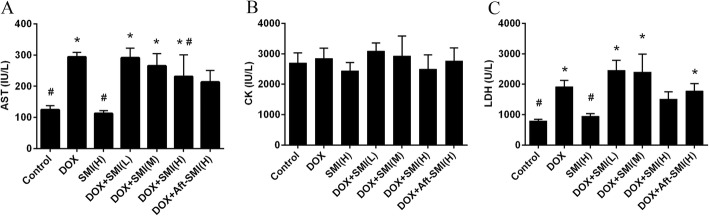
Effect of treatment with SMI and/or DOX on serum levels of AST (**a**), CK (**b**) and LDH (**c**). (Means ± S.D., n = 10). *, significantly different (*P* < 0.05) from respective values in the control group; #, significantly different (*P* < 0.05) from respective values in the DOX group

As the main serum biochemical signs of myocardial damage, serum AST, CK, and LDH of the experimental animals were examined, and the results are shown in Fig. 4. The CK levels of the experimental groups were not significantly different. However, DOX increased AST and LDH levels (*P* < 0.05) in serum. When 5 g/kg of SMI were preinjected, AST and LDH levels reflected the downward trend with no statistically significant difference.

### Histopathological examination

**Fig. 5:**
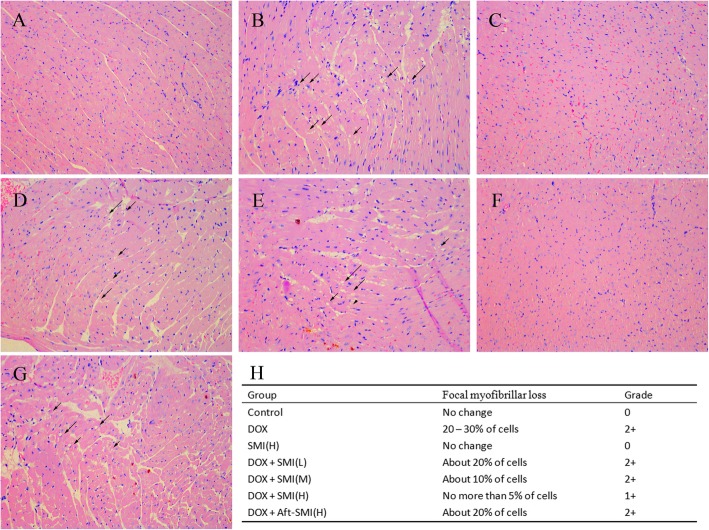
Representative photomicrographs of cardiac tissue from control group (**a**), DOX group (**b**), SM injection group (**c**), co-treatment with DOX and SMI (0.5 g/kg) group (**d**), co-treatment with DOX and SMI (1.5 g/kg) group (**e**), co-treatment with DOX and SMI (5 g/kg) group (**f**) and the co-treatment with DOXand SMI (5 g/kg)) after treating (**g**). Co-treatment DOX and SMI on cardiac damage score (**h**). Cytoplasmic vacuolation and gentle inflammatory infiltration in the DOX treatment were attenuated by SMI. (HE × 200)

Histopathological examination of cardiac tissue was performed as shown in Fig. 5.Slides with heart tissue from control group mice showed regular cell distribution and normal myocardium architecture. Heart sections of DOX-treated mice exhibited vacuolar and gentle inflammatory infiltration. Injection of SMI improved the pathological changes of myocardium as manifested by the decrease in vacuolar degeneration and inflammatory infiltration. As shown in Fig. 5h, the grades scores show a good dose-effect histopathological improvement in the groups of mice pretreated with 0.5, 1.5 and 5 g/kg of SMI.

### Effect of SMI and/or DOX treatment by inflammatory cytokine production

**Fig. 6:**
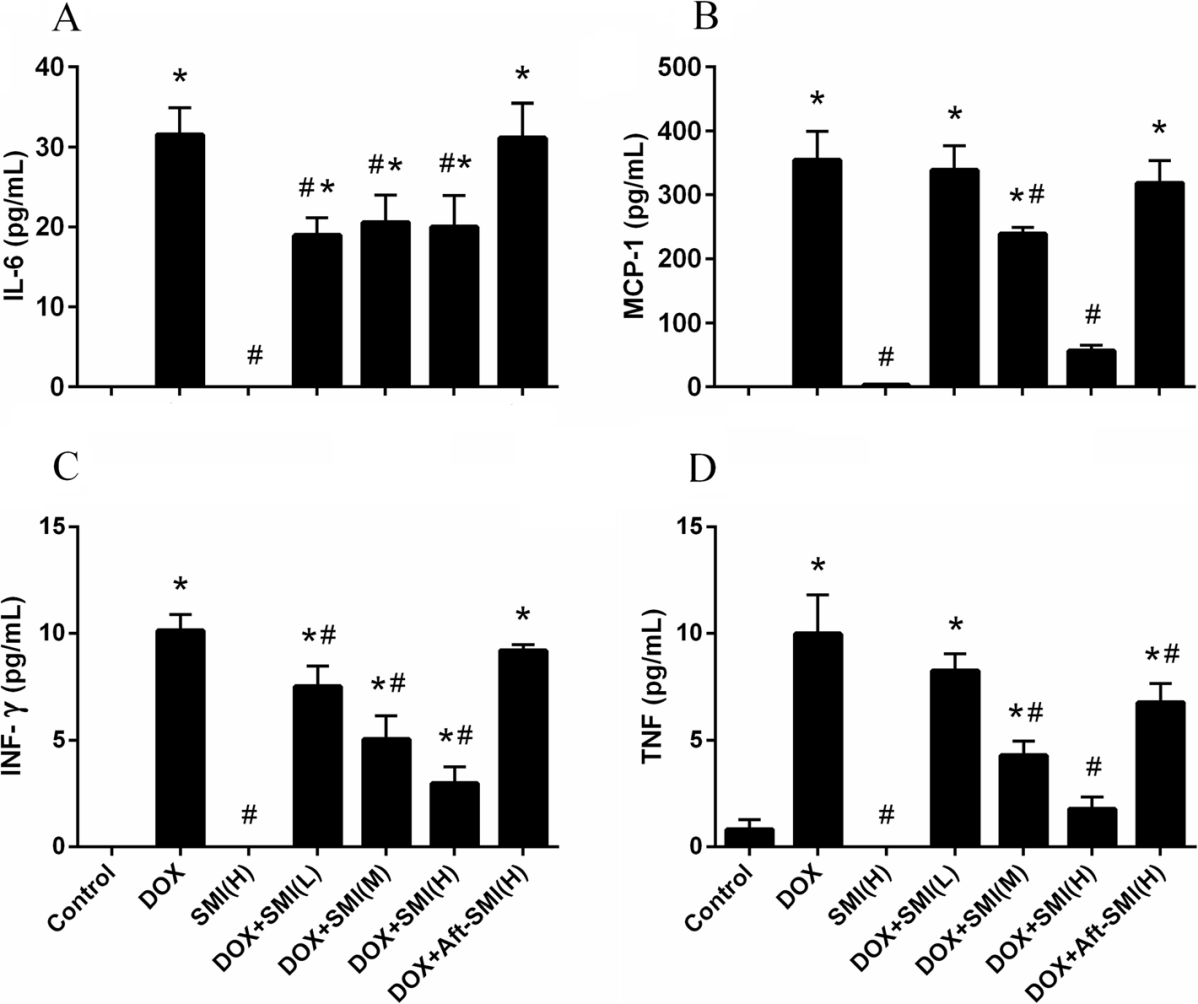
Levels of IL-6 (**a**), MCP-1 (**b**), INF-γ (**c**) and TNF (**d**) in the serum of mice. (Means ± S.D., n = 10). *, significantly different (*P* < 0.05) from respective values in the control group; #, significantly different (*P* < 0.05) from respective values in the DOX group

The result of inflammatory cytokine production is shown in Fig. 6.The result of flow cytometry with the CBA kit showed that the mice treated with DOX showed a significant (*P* < 0.05) increase in IL-6, MCP-1, INF-γand TNF. However, these pro-inflammatory cytokines were hardly detected in the control group. When the SMI were pretreated to mice, these four inflammatory cytokines had a significant down-regulation in the serum. The down-regulation in MCP-1, INF-γ, and TNF showed a dose-dependent relationship.

### Effect of DOX on NOS onset and regulation by SMI

**Fig. 7:**
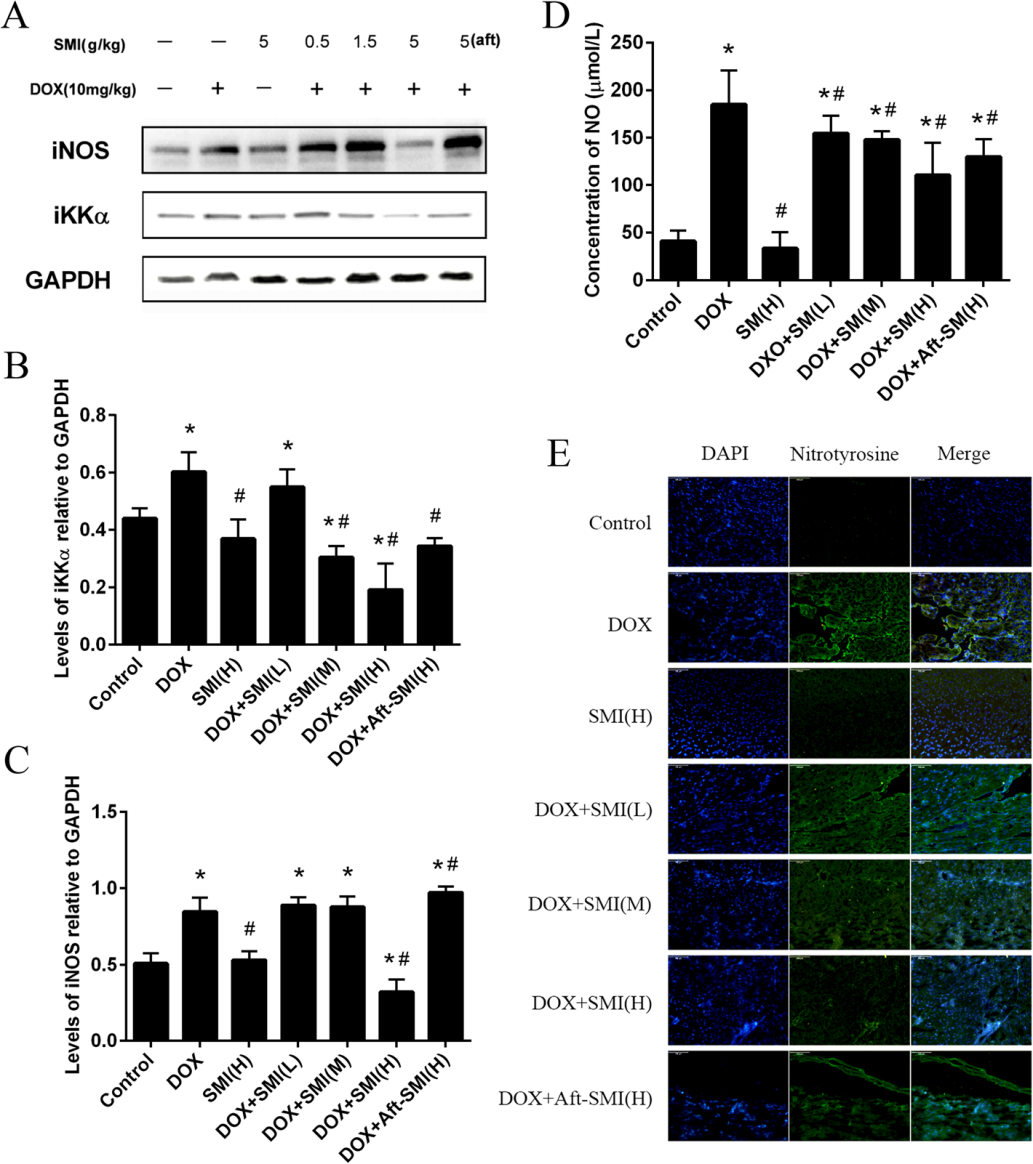
Mice received different treatment in experiment. And IKK-α and iNOS expressions were detected by Western blot analysis into tissue homogenates from mice heart; GAPDH protein expression was used as loading control (**a** – **c**). Effect of DOX on NO release was evaluated by NO level in the serum of mice (**d**). (Means ± S.D., n = 10). *, significantly different (*P* < 0.05) from respective values in the control group;#, significantly different (*P* < 0.05) from respective values in the DOX group. Nitrotyrosine production in heart of ICR mice treat with DOX or SMI (**e**). Frozen myocardial tissue sections were stained with Anti-Nitrotyrosine (green) and nucleus with DAPI (blue) and were determined by immunofluorescence analysis. (Bar 200 μm)

Induces the expression of pro-inflammatory cytokines is mediated by the activation of the NF-κB pathway based on research on the signaling pathway [15, 16]. Our research results showed that DOX induced a significant increase in IKK-α expression (*P* < 0.05). All data points (Fig. 7a–b) demonstrated that SMI was able to decrease the overexpression of IKK-α in heart homogenate. The activation of NF-κB also regulated iNOS transcription. As shown in Fig. 7a and c, the expression of iNOS in the DOX group was significantly increased (P < 0.05) compared to the control group. Pretreatment with 5 mg/kg SMI significantly decreased the expression of iNOS in DOX-induced mice.

Nitrite release directly indicated the production of NO for evaluating the function of heart. Furthermore, the level of NO in serum was measured. NO production was observably increased in the serum of all the mice that were treated with DOX administration(*P* < 0.05, Fig. 7d). In the other hand, SMI significantly decreased (*P* < 0.05) the NO levels in serum compared to the DOX group.

Nitrotyrosine is considered to be an indicator of NO-mediated oxidative inflammatory response. The results of the immunofluorescence analysis suggested that DOX significantly increased nitrotyrosine expression in the mouse heart (Fig. 7e). SMI interruption, especially in the DOX – SMI (H) group, nitrotyrosine in heart tissue was significantly decreased. However, when SMI was treated after DOX injection, nitrotyrosine remained at a high level.

## Discussion

DOX is a chemotherapeutic drug. However, its clinical utility is limited by severe cardiotoxicity [17]. The cause of DOX-induced cardiotoxicity involves various signaling mechanisms, including calcium overloading, free radical stress, dysregulation of iron homeostasis, and mitochondrial dysfunction [18]. In recent findings, DOX induces the expression of NF-κB, an important regulator of genes that join in both immune and inflammatory responses, to cause inflammatory effects on the myocardium and the vasculature [19]. The phenomenon was considered to be the reason for functional impairment in the myocardium. In the present study, the dose of DOX reached at 10 mg/kg weakened the mice, with an altered echocardiographic outcome that is the main cardiac functional parameter. Moreover, cardiac cytoplasmic vacuolation and myofibrillar disarrangement were observed to be the characterized of DOX-induced myocardial damage. These results were related to the increased inflammatory cytokine production and iNOS expression in the heart of mice. In agreement with the previous report [20], our study indicated that the initiation and progression of acute cardiotoxicity induced by DOX is a pivotal factor contributing to inflammation in heart failure [21].SMI is a widely used drug for the treatment of coronary heart disease, viral myocarditis and chronic pulmonary heart disease [22]. The present study demonstrated a dose-dependent cardio-protective effect of SMI against DOX-induced cardiac injury. This observation supports the conclusion that the ability of SMI to counteract DOX-induced cardiac damage is partly due to the improvement of inflammation.

NF-κB plays a key role both in immune and inflammatory responseinduction.NF-κB with an inactive form exists in the cytoplasm that is associated with regulatory protein, which is called inhibitors of κB. Phosphorylation of inhibitors of κB, an important step in NF-κB activation, is mediated by IKK. In the present study, DOX led to a significant increase in NF-κB binding activity. NF-κB regulates a series of obvious pro-inflammatory mediator expression, including TNF-α, IL-1βand NO [23]. The present study demonstrated that DOX increased IKK-α expression and increased NO levels. Conversely, SMI pretreatment reduced IKK-α expression and inhibited the downstream inflammatory cascade. The results were consistent with previous studies that SMI inhibited the over-increased expression of NF-κB to prevent the development of heart disease [24]. Another related study demonstrated that the Shenmai formula, similar to SMI, has been proven to weaken inflammatory damage by blocking the NF-κB pathway in cardiac microvascular endothelial cells [25].

Our data suggested that DOX induced iNOS overexpression and NO level increase. Furthermore, SMI played a constructivec role in iNOS overexpression reduction which was increased by DOX. The results suggested that inhibition of iNOS or anti-NF-κB might be therapeutic targets in cardiotoxicity [26]. Nitrotyrosine is a stable end product of peroxynitrite oxidation, which is produced by the action of reactive nitrogen species such as peroxynitrite anion and nitrogen dioxide. It is used in NO-dependent inflammatory reactions, as an indicator or marker for cell damage, inflammation and NO production. Immunofluorescence analysis revealed a contemporary increase in nitrotyrosine expression in the heart of mice. The result could be explained by the fact that NO from iNOS is reported to function in peroxynitrite outgrowth [27].

Interestingly, we found that SMI can protect the heart and reduce the cardiotoxicity DOX-induced in the prevention group. The myocardial function indexes, such as EF, FS and iNOS expression, in the treatment group showed that SMI ameliorated the cardiotoxicity induced by DOX. SMI reduced the DOX-induced innate immune response, and this behavior led to the inflammatory-related cytokine down-regulation in the NF-κB pathway.

In addition, Sheng-Mai Yin, another traditional Chinese formula that is similar to the SMI formulation and comprises *Panax ginseng*C.A.Mey.*, Ophiopogon japonicus* (Thunb.) Ker Gawl. and *Schisandra chinensis* (Turcz.) Baill.at the ratio of 1:2:1 may protect heart function through the restriction of myocardial fibrosis induced by 15 mg/kg of DOX in rats [28]. The protection of Sheng-Mai Yin against DOX-induced cardiotoxicity was certified by the reduction of myocardial fibrosis, the inhibition of anti-inflammation, the reduction of myocardial fibrosis and the regulation of the cardiac immune microenvironment [28]. The main precondition of a cardioprotective agent for DOX chemotherapy is that it must not influence the antitumor activity of DOX. Together with the Liu study showing that SMI improves the subcellular distributions of DOX to enhance the chemotherapeutic efficacy of DOX in vivo and in vitro [28, 29], studies by Ma and our lab suggest the potential use of SMI in protecting cardiac cells during doxorubicin exposure.

## Conclusion

In conclusion, this article demonstrated that SMI protected the heart by resisting the acute cardiotoxicity induced by doxorubicin. SMI was associated with reduced inflammatory factors caused by doxorubicin-induced acute myocardial damage. The mechanism of protection with SMI involved its ability to down-regulate iNOS and IKK-α in myocardial tissue. Subsequently, SMI calmed acute inflammatory responses and reduced the release of nitrotyrosine in the mouse heart. We hypothesized that the main mechanism of SMI myocardial tissue protection might be associated with the protection of myocardial function and the inhibition of the systemic inflammatory response.

## Data Availability

The datasets used and analysed during the current study are available from the corresponding author on reasonable request.

## Abbreviations

AST: Aspartate aminotransferase
CK: Creatine kinase
CO: Cardiac output
DOX: Doxorubicin
EF: Left ventricular ejection fraction
FS: Fractional shortening
HR: Heart ratio
IKK: Inhibitor of kappa B kinase complex
IL: Interleukin
INF-γ: Interferon-γ
iNOS: Inducible nitric oxide synthase
IκB: An inhibitors of κB
LDH: Lactate dehydrogenase
MCP-1: Monocyte chemotactic protein 1
NF-κB: Nuclear factor Kappa-B
NO: Nitric oxide
ROS: Reactive oxygen species
SMI: Shenmai injection
TNF: Tumor necrosis factor
